# Genetic diversity among *Toxoplasma gondii* strains from different hosts and geographical regions revealed by sequence analysis of GRA5 gene

**DOI:** 10.1186/1756-3305-5-279

**Published:** 2012-12-03

**Authors:** Jia Chen, Zhong-Yuan Li, Dong-Hui Zhou, Guo-Hua Liu, Xing-Quan Zhu

**Affiliations:** 1 State Key Laboratory of Veterinary Etiological Biology, Key Laboratory of Veterinary Parasitology of Gansu Province, Lanzhou Veterinary Research Institute, Chinese Academy of Agricultural Sciences, Lanzhou, Gansu Province 730046, China; 2 College of Veterinary Medicine, South China Agricultural University, Guangzhou, Guangdong Province, 510642, China; 3 College of Veterinary Medicine, Hunan Agricultural University, Changsha, Hunan Province 410128, China

**Keywords:** *Toxoplasma gondii*, *Toxoplasmosis*, GRA5 gene, Genetic diversity, Genotyping

## Abstract

**Background:**

*Toxoplasma gondii* is a highly prevalent protozoan parasite infecting a wide range of animals and humans. The epidemiological and biological diversity of *T. gondii* has resulted in a high genetic variation and unusual population structure in this parasite. This study examined sequence diversity in dense granule 5 (GRA5) gene among *T. gondii* isolates from different hosts and geographical regions.

**Methods:**

The entire genome region of the GRA5 gene was amplified and sequenced from 14 *T. gondii* isolates, and phylogenetic relationship among these *T. gondii* isolates was reconstructed using Bayesian inference (BI) and maximum parsimony (MP) based on the GRA5 sequences.

**Results:**

The complete sequence of the GRA5 gene was 1614 bp in length for strains TgCatBr5 and MAS, but 1617 bp for the other 12 strains. Sequence analysis identified 41 (0–1.7%) variable nucleotide positions among all isolates, with 18 variations of these being in the coding region. Variable positions in the coding region resulted in 11 amino acid substitutions, and a deletion of 3 bp in the strains TgCatBr5 and MAS leading to the deletion of one amino acid. Sequence variations resulted in the existence of polymorphic restriction sites for endonucleases *Aat*II and *Mlu*I, allowing the differentiation of the three major clonal lineage types I, II and III by PCR-RFLP. Phylogenetic analyses using BI and MP supported the clear differentiation of the examined *T. gondii* strains into their respective genotypes.

**Conclusions:**

This study demonstrated the existence of sequence variability in the GRA5 gene sequence among *T. gondii* isolates from different hosts and geographical regions, which allowed the differentiation of the examined *T. gondii* strains into their respective genotypes, suggesting that this highly polymorphic GRA5 locus may provide a new genetic marker for population genetic studies of *T. gondii* isolates.

## Background

*Toxoplasma gondii* is one of the most successful intracellular protozoan parasites belonging to the phylum Apicomplexa with a worldwide distribution, and it is capable of infecting a wide range of warm-blooded animals including birds, humans, livestock and marine mammals [1-6]. In order to adapt to different environments in its hosts, *T. gondii* has evolved a complex life cycle involving the development of asexual forms in various warm-blooded hosts, with the occurrence of sexual replication only in the cat gut [2], as well as established different modes of transmission including the ability of transmission between intermediate hosts through carnivorous or omnivorous feeding [7]. Such an adaptation to various ecological systems has given rise to a highly genetic variation and unusual population structure in the parasite [8,9].

The majority of *T. gondii* isolates in North America and Europe have been classified into four major clonal lineage types (I, II, III and 12) [10-13]. Furthermore, *T. gondii* strains corresponding to different genotypes within populations show an unevenly geographical distribution, and different clonal types among isolates are responsible for different toxoplasmosis in humans and animals [7,14]. Therefore, our thorough knowledge of genetic diversity of *T. gondii* is central to better understand epidemiological patterns and pathogenicity, as well as explore the new strategies for vaccination, treatment, or diagnosis of toxoplasmosis.

*T. gondii* dense granule 5 (GRA5) is a soluble molecule that can be secreted to the parasitophorous vacuole (PV) and may have a critical function in parasite-host interaction [15,16]. In addition, GRA5 can be used as strain-specific antigen to serotype *T. gondii* strains by enzyme-linked immunosorbent assay (ELISA) [17,18]. These encouraging findings have stimulated the present study, the objectives of which were to examine sequence diversity in GRA5 gene among *T. gondii* isolates from different hosts and geographical regions, and to assess whether the GRA5 gene sequence may be used as a new marker for population genetic studies of *T. gondii* isolates.

## Methods

### *T. gondii* isolates

A total of 14 *T. gondii* isolates originating from different hosts and geographic locations were used for analysis in this study (Table 1). These *T. gondii* isolates had been genotyped and genomic DNA (gDNA) prepared as described previously [19-22].

**Table 1:** **Details of *Toxoplasma gondii *isolates used in the present study**

**Strain**	**Host**	**Geographical origin**	Genotype^*^
GT1	Goat	United States	Reference, Type I, ToxoDB #10
RH	Human	France	Reference, Type I, ToxoDB #10
PTG	Sheep	United States	Reference, Type II, ToxoDB #1
CTG	Cat	United States	Reference, Type III, ToxoDB #2
TgCatBr5	Cat	Brazil	Reference, ToxoDB #19
MAS	Human	France	Reference, ToxoDB #17
TgPNY	Pig	Luying, Henan, China	Type I, ToxoDB #10
S10	Tree sparrow	Fuzhou, Fujian, China	Type I, ToxoDB #10
Prugniaud (PRU)	Human	France	Type II, ToxoDB #1
QHO	Sheep	Huzhu, Qinghai, China	Type II, ToxoDB #1
JSEM1	House sparrow	Emin, Xinjiang	Type II variant, ToxoDB #3
TgC7	Cat	Guangzhou, Guangdong, China	ToxoDB #9
PYS	Pig	Panyu, Guangdong, China	ToxoDB #9
ZC	Pig	Zengcheng, Guangdong	ToxoDB #9

* based on genotyping results of Zhou *et al.* (2009, 2010), Su *et a*l. (2010) and Huang *et al*. (2012).

### PCR amplification

To obtain amplicons of the genomic sequence of the GRA5 gene, gDNA of individual *T. gondii* isolates was used as a template and amplified by PCR with a pair of oligonucleotide primers designated CJA (forward primer), 5’-AGAAACTGATGCTGCTATA-3’ and CJB (reverse primer), 5’-TCTGAGCATCTTACTGGTG-3’ based on the nucleotide sequence of GRA5 gene available in the ToxoDB database (TGGT1_037870). The amplification reaction was carried out in a volume of 25 μl containing 10 mM Tris–HCl (pH 8.4), 50 mM KCl, 3 mM MgCl_2_, 250 μM each of dNTP, 0.2 μM of each primer, 100–200 ng of template DNA, and 0.25 U La *Taq* polymerase (TaKaRa). Amplification of DNA samples from individual isolates was carried out in a thermocycler (Bio-Rad, Hercules, CA, USA) under the following conditions: denaturation at 94°C for 10 min (initial denaturation), followed by 35 cycles consisting of 94°C for 30 sec (denaturation), 54°C for 30 sec (annealing), 72°C for 2 min (extension), and a final extension step was at 72°C for 10 min. Confirmation of successful PCR amplifications was carried out by electrophoresis on a 1% (w/v) agarose gel, stained with GoldenView™ and photographed using a gel documentation system (UVP GelDoc-It™ Imaging System, Cambridge, UK).

### Sequencing of the GRA5 amplicons

The GRA5 PCR products were purified using the spin columns according to the manufacturer's recommendations (Wizard™ PCR-Preps DNA Purification System, Promega, USA), ligated with pMD 18-T vector (TaKaRa), and then transformed into the JM109 competent cells (Promega, USA). Following the screening by PCR amplification and enzymatic digestion, the positive colonies were sequenced by Shanghai Songon Biological Engineering Biotechnology Company with ABI 377 automated DNA sequencer (BigDye Terminator Chemistry).

### Sequence analysis and phylogenetic reconstruction

The obtained GRA5 gene sequences from different *T. gondii* strains were aligned using Multiple Sequence Alignment Program, Clustal X 1.83 [23], and sequence variation was determined among the examined *T. gondii* strains. Phylogenetic re-constructions based on the complete sequences of GRA5 gene among different *T. gondii* strains was performed using two methodologies, namely Bayesian inference (BI) and maximum parsimony (MP). BI analyses were conducted with four independent Markov chains run for 10,000,000 metropolis-coupled MCMC generations, sampling a tree every 10000 generations in MrBayes 3.1.1 [24]. The first 250 trees were omitted as burn-in and the remaining trees were used to calculate Bayesian posterior probabilities (PP). MP analysis was performed using PAUP* 4.0b10 [25], with indels treated as missing character states. A total of 1,000 random addition searches using tree bisection-reconnection (TBR) branch swapping were performed for each MP analysis. Bootstrap probability (BP) was calculated from 1,000 bootstrap replicates with 10 random additions per replicate in PAUP.

### Characterization of *T. gondii* DNA isolates by PCR-RFLP

To assess whether GRA5 gene sequence is suitable for genotyping of *T. gondii* isolates, *T. gondii* isolates representing three dominant genotypes (Type I, Type II, Type III) were characterized by the PCR–RFLP method described previously [26,27]. In brief, the PCR products were digested by restriction enzymes *Aat*II and *Mlu*I and incubated at 37°C for 4 h according to the manufacturer’s instructions. The restriction fragments were resolved in 1% agarose gel, stained with GoldenView™ and photographed using a gel documentation system (UVP GelDoc-It™ Imaging System, Cambridge, UK).

## Results

### Sequence analysis

The GRA5 amplicons produced a single product of approximately 1600 bp in length on agarose gel for all 14 examined *T. gondii* strains, and then the amplicons of all isolates were sequenced. The complete genome sequence of GRA5 gene was 1614 bp in length for the strains TgCatBr5 and MAS, but 1617 bp in length for the other 12 strains. The alignment of all 14 sequences revealed nucleotide polymorphisms at 41 positions (0–1.7%) (Additional file 1: Table S1). Of these variable nucleotide positions, there were 38 nucleotide substitutions in all the 14 sequences, and two deletions of 3 bp in the sequence of both strains TgCatBr5 and MAS. Among the entire coding region of the GRA5 gene, there were 18 variable positions showing 3.24% overall sequence variation, and thus the most variable regions were at the coding region of the gene. Amino acid sequence analysis of all strains showed the presence of 10 substitutions and one deletion in strains TgCatBr5 and MAS, respectively (Additional file 2: Table S2).

**Figure 1:**
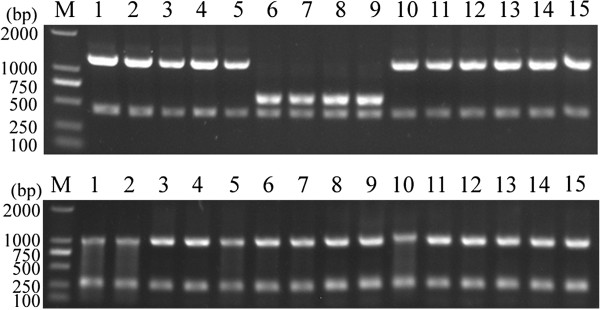
**PCR-RFLP analysis of GRA5 genomic region of *Toxoplasma gondii *isolates in 1% agarose gel using restriction endonucleases *Aat *II (upper) and *Mlu *I (bottom).** Lane M represents DNA size marker 2000. Lanes 1–15 represent *T. gondii* Type I (GTI, RH, TgPNY, S10), Type II (PRU, QHO, PTG, JSEM1), Type III (CTG) strains and other strains (TgCatBr5, MAS, TgC7, PYS and ZC), respectively. Refer to Table 1 for isolate information.

Analysis of sequence polymorphisms in the GRA5 gene among the three clonal genotypes (strains RH/GTI, PRU/QHO/PTG, CTG) revealed the existence of polymorphic restriction sites. One and two *Aat*II restriction sites were observed for Type I and Type II strains, respectively; and three, three and one *Mlu*I restriction sites were observed for Type I, II, III strains, respectively (at positions 419, 1038, 292, 565, 632). Using PCR-RFLP method, digestion of the amplification products with *Aat*II and *Mlu*I allowed the differentiation of strains representing genotypes I, II, and III (Figure 1).

### Phylogenetic analysis of *T. gondii* strains based on GRA5 sequences

**Figure 2:**
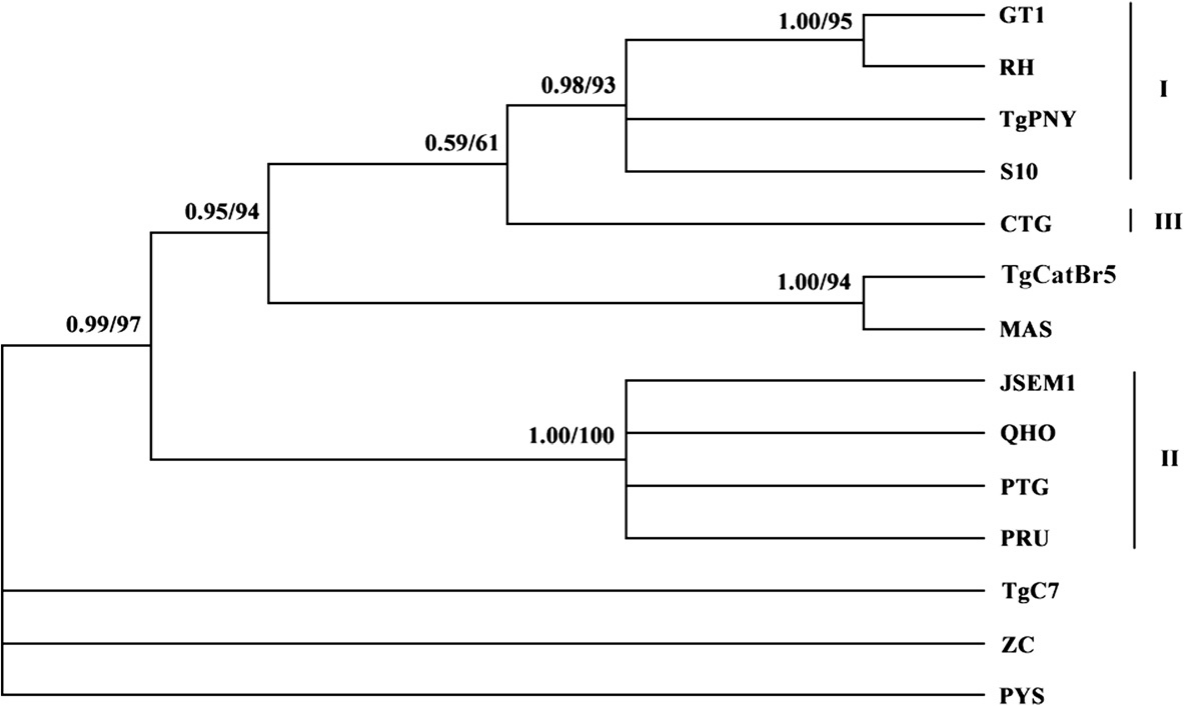
**Phylogram of 14 *Toxoplasma gondii *strains determined by analysis of the entire sequences of the GRA5 genomic region.** The high genetic divergence of GRA5 gene revealed three major clusters (denoted by I, II, and III). The tree was built by Bayesian inference (BI) and maximum parsimony (MP) analysis. The numbers along branches indicate bootstrap values resulting from different analyses in the order: BI/MP.

By phylogenetic reconstruction based on GRA5 sequence data of all 14 strains, we have obtained the phylogram (Figure 2). Phylogenetic analysis revealed three major clusters, which correspond to classical genotypes (I, II, III) respectively, and Type III are clustered more closely with Type I than with other strains. Moreover, the strains TgC7, PYS and ZC were separated from classical genotypes. All the strains belonging to Type I clustered together, including PNY, S10 and typical strains (GTI, RH), but the strains TgPNY and S10 pertain to Type I. Atypical strains MAS and TgCatBr5 were phylogenetically linked to Type III. Additionally, four strains, including three classical strains PRU, PTG, QHO, and a new isolated strain JSEM1 previously identified by Huang [22] were grouped into Type II cluster phylogenetically.

## Discussion

In the last few decades, a variety of different loci have been analyzed based on sequencing of housekeeping genes, antigens and neutral introns [28]. This sequence-based analysis can provide an ideal approach for displaying the complete genetic diversity including small insertions and deletions (e.g. indels) and single nucleotide polymorphisms (SNPs) that could then be used to determine more precise relationships between different *T. gondii* isolates and possibly even facilitate phylogenetic classifications [8].

In the present study, we determined the entire genome sequence of the GRA5 locus for 14 *T. gondii* strains from different geographical locations and host origins, and examined genetic diversity between *Toxoplasma* strains based on GRA5 gene sequences. The genome sequence of the GRA5 locus was found to be considerably polymorphic having 41 variable positions among the 14 examined isolates; by contrast, only 18 nucleotides were variable in the coding region, which again illustrated that direct sequencing of genomic regions can capture the full genetic diversity [8]. The analyses of sequence variations in nucleotides and amino acids in antigenic regions among individual isolates have shown the high ratio of non-synonymous to synonymous amino acid changes, suggesting that GRA5 is undergoing positive selection, together with several polymorphic GRA antigens including GRA3, GRA6 and GRA7 [29-31]. Sequence variations in other loci including GRA3 [29] and GRA7 [31], SAG2 [32] and SAG3 [33] have been studied, and have shown slightly lower polymorphisms than in GRA5, but GRA6 sequence [30] is more polymorphic than GRA5.

Moreover, by digestion of PCR products using endonucleases *Aat*II and *Mlu*I, the three major clonal lineages (Type I, II and III) can be differentiated, which suggests that the GRA5 locus may represent a potential new genetic marker for PCR-RFLP genotyping of *T. gondii* strains. However, some strains isolated from China including TgC7, PYS, ZC, S10, and TgPNY, and atypical strains TgCatBr5 and MAS cannot be differentiated from the three major lineage types (Figure 1), suggesting that PCR-RFLP genotyping with a single locus is not enough for the identification of non-clonal types. Phylogenetic analysis using BI and ML based on the genomic sequences of the GRA5 locus showed that the three major clonal lineages clustered into their respective genotypes (groups) separately, and Type III was more closely related to Type I. This result is consistent with that of some previous studies using GRA6 and AK genotyping [30,34].

## Conclusion

The present study demonstrated the existence of sequence variability in the GRA5 locus among *T. gondii* isolates from different hosts and geographical regions, which allowed the differentiation of the examined *T. gondii* strains into their respective genotypes. These findings suggest that this highly polymorphic GRA5 locus may provide a new genetic marker for population genetic studies of *T. gondii* isolates. Further studies are needed to test this possibility when more *T. gondii* strains originating from different countries/regions and different hosts, in particular from wild animals, become available.

## Competing interests

The authors declare that they have no competing interests.

## Authors’ contributions

XQZ and JC conceived and designed the study, and critically revised the manuscript. JC and ZYL performed the experiments, analyzed the data and drafted the manuscript. DHZ and GHL helped in study design, study implementation and manuscript revision. All authors read and approved the final manuscript.

## Supplementary Material

Additional file 1 **Table S1.** Nucleotide polymorphism of the GRA5 gene genomic region among *Toxoplasma gondii* isolates. Click here for file

Additional file 2 **Table S2.** Variation in the predicted amino acid sequences of the GRA5 gene coding region among *Toxoplasma gondii* isolates. Click here for file
