# Supplementary material for: Genetic diversity among Toxoplasma gondii strains from different hosts and geographical regions revealed by sequence analysis of GRA5 gene
Source: Parasit Vectors. 2012 Dec 3;5:279. doi: 10.1186/1756-3305-5-279 (PMC3533945; doi:10.1186/1756-3305-5-279)
Supplement: Additional file 1 — Table S1. Nucleotide polymorphism of the GRA5 gene genomic region among Toxoplasma gondii isolates. [file 1756-3305-5-279-S1.doc]

**Table S1 Nucleotide polymorphism of the GRA5 gene genomic region among *Toxoplasma gondii* isolates**

| **Strains** | **Positions of variable nucleotides** | | | | | | | | | | | | | | | | | | | | | | | |
| --- | --- | --- | --- | --- | --- | --- | --- | --- | --- | --- | --- | --- | --- | --- | --- | --- | --- | --- | --- | --- | --- | --- | --- | --- |
|  | **17** | **127** | **136** | **164** | **354** | **383** | **392** | **472** | **634** | **641** | **642** | **654** | **663** | **674** | **680** | **687** | **701** | **702** | **703** | **708** | **714** | **761** | **789** | **798** |
| RH | A | G | G | A | T | C | G | A | G | G | T | G | C | A | C | A | C | A | A | A | A | C | C | T |
| GT1 | A | G | G | A | T | C | G | A | G | G | T | G | C | A | C | A | C | A | A | A | A | C | C | T |
| PNY1 | A | G | G | G | T | C | G | A | G | G | T | G | C | A | C | A | C | A | A | A | A | C | C | C |
| S10 | A | G | G | G | T | C | G | A | G | G | T | G | C | A | C | A | C | A | A | A | A | C | C | T |
| QHO | A | G | A | G | T | T | G | A | G | A | C | G | C | T | G | A | C | A | A | G | G | C | C | T |
| PRU | A | G | A | G | T | T | G | A | G | A | C | G | C | T | G | A | C | A | A | G | G | C | C | T |
| PTG | A | G | A | G | T | T | G | A | G | A | C | G | C | T | G | A | C | A | A | G | G | C | C | T |
| JSEM1 | A | G | A | G | T | T | G | A | G | A | C | G | C | T | G | A | C | A | A | G | G | C | C | T |
| CatBr5 | A | A | A | G | T | C | G | A | G | G | T | G | T | G | C | G | - | - | - | A | A | A | C | T |
| MAS | A | A | A | G | T | C | G | A | G | G | T | G | T | G | C | G | - | - | - | A | A | A | C | T |
| TgC7 | A | G | A | G | C | C | T | G | G | A | C | G | C | G | C | G | C | A | A | A | A | C | C | T |
| PYS | A | G | A | G | T | C | T | G | G | A | C | G | C | G | C | G | C | A | A | A | A | C | C | T |
| ZC | A | G | A | G | T | C | T | G | A | A | C | G | C | G | C | G | C | A | A | A | A | C | C | T |
| CTG | A | G | A | G | T | C | G | A | G | G | T | C | C | G | C | G | C | A | A | A | A | C | T | T |

Dashes (-) indicate deletions.

**Table S2** (continued)

| **Strains** | **Positions of variable nucleotides** | | | | | | | | | | | | | | | | | |
| --- | --- | --- | --- | --- | --- | --- | --- | --- | --- | --- | --- | --- | --- | --- | --- | --- | --- | --- |
|  | **831** | **841** | **973** | **979** | **1010** | **1023** | **1042** | **1046** | **1091** | **1137** | **1330** | **1355** | **1384** | **1391** | **1402** | **1489** | **1571** | **1577** |
| RH | A | A | C | G | T | T | C | T | C | C | T | A | T | A | G | A | C | C |
| GT1 | A | A | C | G | T | T | C | T | C | C | T | A | T | A | G | A | C | C |
| TgPNY | A | A | C | G | C | C | C | T | C | T | T | A | T | G | G | A | C | T |
| S10 | A | A | C | G | T | T | C | T | C | T | T | A | T | A | G | A | C | T |
| QHO | G | G | G | G | T | T | T | T | G | T | G | A | T | A | A | G | G | T |
| PRU | G | G | G | G | T | T | T | T | G | T | G | A | T | A | A | G | G | T |
| PTG | G | G | G | G | T | T | T | T | G | T | G | A | T | A | A | G | G | T |
| JSEM1 | G | G | G | G | T | T | T | C | G | T | G | G | C | A | A | G | G | T |
| TgCatBr5 | A | G | C | G | T | T | C | T | C | T | T | A | T | A | G | A | C | T |
| MAS | A | G | C | G | T | T | C | T | C | T | T | A | T | A | G | A | C | T |
| TgC7 | G | G | C | A | T | G | C | T | C | T | T | A | T | A | G | A | G | T |
| PYS | G | G | C | A | T | G | C | T | C | T | T | A | T | A | G | A | G | T |
| ZC | G | G | C | A | T | G | C | T | C | T | T | A | T | A | G | A | G | T |
| CTG | A | A | C | G | T | T | C | T | C | T | T | A | T | A | G | A | C | T |

Dashes (-) indicate deletions.
