# Supplementary material for: Genetic diversity among Toxoplasma gondii strains from different hosts and geographical regions revealed by sequence analysis of GRA5 gene
Source: Parasit Vectors. 2012 Dec 3;5:279. doi: 10.1186/1756-3305-5-279 (PMC3533945; doi:10.1186/1756-3305-5-279)
Supplement: Additional file 2 — Table S2. Variation in the predicted amino acid sequences of the GRA5 gene coding region among Toxoplasma gondii isolates. [file 1756-3305-5-279-S2.doc]

**Table S2 Predicted amino acid sequence variation of the GRA5 gene coding region among *Toxoplasma gondii* strains.**

| **Strains** | **Positions of amino acid (deduced)** | | | | | | | | | | | | |
| --- | --- | --- | --- | --- | --- | --- | --- | --- | --- | --- | --- | --- | --- |
|  | **31** | **35** | **38** | **42** | **44** | **46** | **51** | **53** | **55** | **71** | **80** | **83** | **94** |
| RH | V | G | S | R | R | Q | Q | E | N | H | A | V | K |
| GT1 | V | G | S | R | R | Q | Q | E | N | H | A | V | K |
| TgPNY | V | G | S | R | R | Q | Q | E | N | H | A | A | K |
| S10 | V | G | S | R | R | Q | Q | E | N | H | A | V | K |
| QHO | T | G | S | W | G | Q | Q | G | S | H | A | V | R |
| PRU | T | G | S | W | G | Q | T | G | S | H | A | V | R |
| PTG | T | G | S | W | G | Q | Q | G | S | H | A | V | R |
| JSEM1 | T | G | S | W | G | Q | Q | G | S | H | A | V | R |
| TgCatBr5 | V | G | F | G | R | R | - | E | N | N | A | V | K |
| MAS | V | G | F | G | R | R | - | E | N | N | A | V | K |
| TgC7 | T | G | S | G | R | R | Q | E | N | H | A | V | R |
| PYS | T | G | S | G | R | R | Q | E | N | H | A | V | R |
| ZC | T | G | S | G | R | R | Q | E | N | H | A | V | R |
| CTG | V | A | S | G | R | R | Q | E | N | H | V | V | K |

Dashes (-) indicate deletions.
